# 3D Concentric Electrodes-Based Alternating Current Electrohydrodynamics: Design, Simulation, Fabrication, and Potential Applications for Bioassays

**DOI:** 10.3390/bios12040215

**Published:** 2022-04-06

**Authors:** Raphaela K. S. Silva, Sakandar Rauf, Ming Dong, Liang Chen, Hakan Bagci, Khaled N. Salama

**Affiliations:** 1 Sensors Laboratory, Advanced Membranes & Porous Materials Centre (AMPMC), Computer, Electrical, and Mathematical Sciences and Engineering (CEMSE) Division, King Abdullah University of Science and Technology (KAUST), Thuwal 23955-6900, Saudi Arabia; raphaelakethlim.souzasilva@kaust.edu.sa (R.K.S.S.); sakandar.rauf@kaust.edu.sa (S.R.); 2 Electrical and Computer Engineering (ECE) Program, Computer, Electrical, and Mathematical Science and Engineering (CEMSE) Division, King Abdullah University of Science and Technology (KAUST), Thuwal 23955-6900, Saudi Arabia; ming.dong@kaust.edu.sa (M.D.); liang.chen@kaust.edu.sa (L.C.); hakan.bagci@kaust.edu.sa (H.B.)

**Keywords:** alternating current electrohydrodynamics (ac-EHD), concentric electrodes, 3D electrodes, electric field simulation, fluid micromixing, bioassays

## Abstract

Two-dimensional concentric asymmetric microelectrodes play a crucial role in developing sensitive and specific biological assays using fluid micromixing generated by alternating current electrohydrodynamics (ac-EHD). This paper reports the design, simulation, fabrication, and characterization of fluid motion generated by 3D concentric microelectrodes for the first time. Electric field simulations are used to compare electric field distribution at the electrodes and to analyze its effects on microfluidic micromixing in 2D and 3D electrodes. Three-dimensional devices show higher electric field peak values, resulting in better fluid micromixing than 2D devices. As a proof of concept, we design a simple biological assay comprising specific attachment of streptavidin beads onto the biotin-modified electrodes (2D and 3D), which shows ~40% higher efficiency of capturing specific beads in the case of 3D ac-EHD device compared to the 2D device. Our results show a significant contribution toward developing 3D ac-EHD devices that can be used to create more efficient biological assays in the future.

## 1. Introduction

Fluid flow control using different microdevices has shown great importance for the development of biosensors. As manufacturing processes achieve accurate micron-sized sensors and actuators, the demand to manipulate fluids in small volumes has been reported in many fields, including biotechnology [1,2,3], nanomaterials synthesis [4], and lab-on-a-chip [5,6]. Once dimension shrinks, fluid behavior differs from what is observed in the macro scale, surface forces dominate, and traditional pressure-driven flows suffer from large viscous stress [7]. In this context, electrohydrodynamic (EHD) forces arising from an external electric field provide an efficient means of microflow control [8]. The use of electrical driving forces is well known in other electrokinetic phenomena, such as dielectrophoresis [9], in which polarizable particles move in response to a non-uniform electric field. The mechanism allows the selective concentration of particles but is highly dependent on the particle’s electrical properties and dimensions [9]. Alternating current electrohydrodynamics (ac-EHD) is usually preferred over its direct current counterpart [10]. The main advantage stems from operating at lower voltages, making it suitable for biological applications and portable systems. For example, one drawback in biosensors development is the transport of analytes from the bulk fluid to a transducer surface. The diffusive process takes up to several hours and has been recognized as one of the main factors hampering the achievement of lower detection limits in biosensors [11]. However, the application of ac-EHD induces a fluid motion that accelerates mass transport and enhances the capture of specific analytes, reducing the sensing time up to 13 times in some applications [12]. Electrical driving forces have previously been demonstrated to enhance the analyte transport of many bioparticles to the transducer surface [13,14,15,16,17]. Furthermore, the induced shear forces also enhance specific adsorption through the displacement of weekly bounded non-specific analytes [18]. Going beyond biosensors, examples of the applications of EHD include heat transfer [19,20], the formation of nanoparticle monolayers [21], polymer nanoparticle synthesis [22], and driving force to chemical reactions [23].

One unique feature of ac-EHD is the ability to tune fluid motion by selecting electrode geometry and ac parameters (voltage and frequency). Initially, it was observed that symmetric planar electrodes do not engage directional fluid flow [24], while asymmetric electrodes could generate directional pumping [25]. Bearing on this, different geometries have been designed to create different fluid movement patterns. For instance, arrays of planar interdigitated electrodes are usually employed to pump the fluid through a microchannel [8], while planar circular electrodes are more suitable for fluid micromixing purposes [16,18,26,27]. In the same context of enhancing electrodes’ performance by manipulating their geometry, three-dimensional electrodes have found successful applications in areas such as energy storage [28], neural probes [29], and biosensing [30], primarily due to their larger surface area. However, 3D concentric electrodes have not been studied for their fluid motion pattern and potential use for biosensor applications.

To investigate the fluid patterns, ac-EHD characterization often involves fluorescent beads [18,31]. With imaging techniques such as particle image velocimetry [31] and image processing [32], it is possible to estimate the bead’s velocity and characterize the fluid motion. However, previous studies only report an average velocity when discussing their results, while a new approach by dividing the velocity measurements into different time intervals can give more insightful data to describe the ac-EHD phenomenon.

Herein, for the first time, we designed and fabricated 3D ac-EHD concentric asymmetric microelectrodes and compared fluid micromixing performance against 2D electrodes in a model bioassay format. First, the effect of electrode spacing on ac-EHD induced fluid micromixing was investigated among different 2D asymmetric circular electrodes using fluorescence microscopy. Three electrodes with spacings of 50 µm, 200 µm, and 1000 µm between inner and outer electrodes were studied. To investigate the ac-EHD driven fluid motion, fluorescent beads dispersed in a buffer solution were used. The beads’ movement was captured on video, and single-particle tracking was performed to estimate their maximum velocity. We report a new strategy of analyzing the average bead velocity in segments rather than an overall average, as previously reported [18,33]. This new strategy gives in-depth information about fluid micromixing efficiency with time in different ac-EHD devices. Also, this study shows that the dielectrophoresis phenomenon is highly dependent on the distance between the inner and outer electrodes in the case of concentric electrodes. To validate our results, electrical field distribution simulations were also performed. Finally, we introduce a 3D variation of a 1000-µm spacing device containing a microtip centered in the inner electrode. For the 3D case, we studied the impact of its larger surface area, comparing the performance of both 2D and 3D electrodes in a proof-of-concept fluorescence assay.

## 2. Materials and Methods

### 2.1. Design and Fabrication of Microdevices

**Figure 1:**
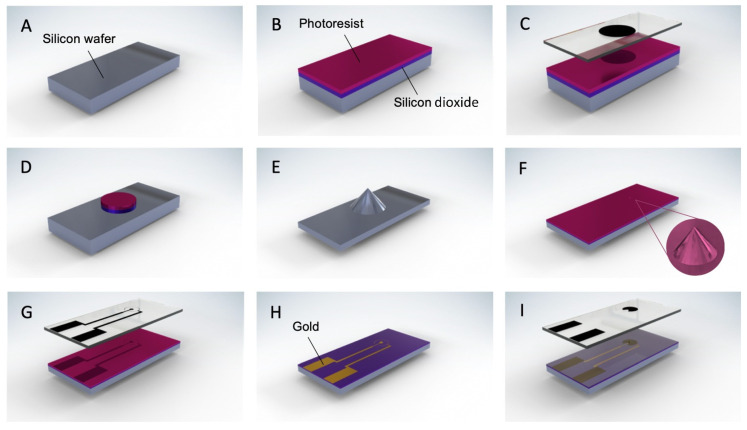
Schematic of device fabrication. The bare silicon wafer in (**A**), is covered by 1 μm of silicon dioxide and 1.6 μm of AZ5214E photoresist (**B**). UV exposure through the first chrome mask (**C**) patterns the wafer with photoresist circles after developing. The non-covered oxide is etched to reveal the same pattern (**D**). Together, oxide and photoresist work as a hard mask during DRIE to obtain the tip structures (**E**). Following, a 1-μm layer of silicon dioxide is deposited by thermal means, along with 2 μm of AZ2020 (**F**). UV exposure through the second chrome mask (**G**) followed by developing, transfers the electrode patterns to the wafer. Ten nanometers of titanium and 200 nm of gold are deposited by electron beam evaporation and overnight lift-off removes the excess metal out of the pattern (**H**). A final 2-μm layer of AZ2020 photoresist is deposited and UV exposure through the third chrome mask (**I**) insulates the whole wafer except the pads and electrode area.

Figure 1 shows the fabrication scheme to prepare 2D and 3D electrodes. In this study, three different geometries were fabricated, where the distance between the inner circular electrode and outer ring electrode varies between 50 μm, 200 μm, and 1000 μm. All the other parameters, such as the diameter of the inner electrode and thickness of the outer electrode, were kept the same for the three designs. As previously mentioned, the electrode geometry determines the induced motion pattern. In our case, the asymmetry between circular and ring electrodes guarantees a lateral motion from the inner to the outer electrode.

The designs were made in Tanner L-edit and transferred to a chrome mask by direct laser writing (Heidelberg Instruments uPG501, Heidelberg, Germany). For the 3D devices, the silicon wafers were initially cleaned for 20 min in a solution of sulfuric acid and hydrogen peroxide mixture (piranha cleaning). Silicon dioxide was deposited by plasma-enhanced chemical vapor deposition (PECVD), and a 1.6-μm-thick layer of positive photoresist (AZ5214E) was spin-coated onto the wafer, followed by a 2-min soft baking at 110 °C. UV exposure through a mask aligner was followed by 1 min developing in AZ726 to reveal a pattern of photoresist circles of 150 μm. The oxide layer not covered by the photoresist was removed by plasma etching to obtain the same pattern. Afterward, a non-passivated deep reactive ion etching (DRIE) was performed for 50 min. By applying SF6 alone and using the circle patterns as a hard mask, we obtained tip structures in a cone-like shape. To insulate these structures, 1 μm of silicon dioxide was deposited by thermal means (Figure 1A–E).

Second photolithography, now with AZ2020, transfers the electrode pattern on top of the etched wafer. The negative photoresist was spin-coated onto the wafer, followed by a 1 min baking at 110 °C. After UV exposure, post-baking was carried out for 1 min at 100 °C followed by a 1 min development in AZ726 to reveal the electrode pattern. To create conductive pathways, 10 nm of titanium and 200 nm of gold were deposited by e-beam evaporation. This process was followed by acetone lift-off carried overnight and sonication to remove the released metal (Figure 1F–H).

Finally, third photolithography step was performed to expose the electrodes and connecting pads and cover the remaining wafer (Figure 1I). This step applies the same AZ2020 photoresist and, therefore, the same recipe mentioned above was used. In all three processes, the wafers were rinsed in de-ionized water and dried by nitrogen gas. The residual photoresist was removed by 30 s of oxygen plasma cleaning. For 2D devices, a similar fabrication was employed, skipping only the part corresponding to the tip fabrication. All the ac-EHD devices were imaged using Quanta 3D FEG SEM/FIB scanning electron microscope (SEM) with an accelerating voltage of 10 kV.

### 2.2. Ac-EHD Experiments Setup

**Figure 2:**
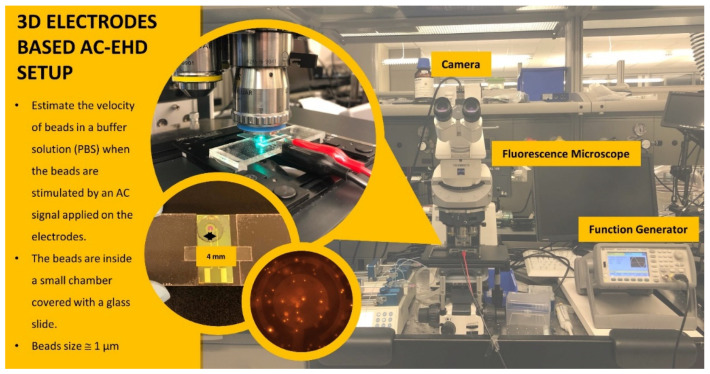
Schematic of the experimental setup for the characterization of 2D and 3D ac-EHD devices.

The electrodes were connected by their gold pads to a function generator (Agilent 33510B waveform Generator, Agilent Technologies, Santa Clara, CA, USA), and positioned under a fluorescence microscope equipped with a dual bandpass filter set (ET-FITC/CY3) and a 40× objective (Figure 2). A polydimethylsiloxane (PDMS) chamber of 0.4 mm thickness and 4 mm diameter aperture was placed on top of the electrodes to create a reservoir able to accommodate 15 µL of the bead solution (carboxyl functionalized Suncoast yellow fluorescent microsphere, average size 0.97 μm, ~2 × 10^9^ beads per ml, dispersed in 1 mM of phosphate buffer saline (PBS)). The particle motion was captured on video by a camera (Blackfly S, BFS-U3-70S7C-C, 51 fps) connected on top of the microscope. The beads’ speed is estimated using NIH ImageJ and Trackmate plugin [34]. For biological assay experiments, 2D and 3D gold electrodes were first incubated with a 1-mM solution of cystamine dihydrochloride (Sigma Aldrich, St. Louis, MI, USA) for two hours [35]. After washing with de-ionized water and drying with nitrogen, electrodes were incubated in 3 mg/mL solution of EZ-Link sulfo-NHS-LC-biotin (Thermo Scientific, Waltham, MA, USA) in PBS for 3 h and finally washed with PBS to remove any unbound reagent [18]. Finally, the electrodes were incubated in a solution containing ~5 × 10^8^ streptavidin-coated dragon green fluorescent microspheres of an average size of 1.04 µm (Bangs laboratories, Fishers. US), and ac-EHD parameters used for 2D and 3D devices were 100 Hz, 4V_pp_. After that, the devices were washed with PBS, and imaging was carried out using a fluorescence microscope equipped with an FITC filter cube and a CCD camera (GS3-U3-91S6M-C, FLIR) for the excitation and imaging of the fluorescent beads.

### 2.3. Electric Field Simulations

Since the fluid motion is strongly related to the electric field’s magnitude and direction, we performed 3D simulations to investigate the electric field distribution inside the electrodes and analyze its effect on microfluidic motion. All the simulations were conducted with the 3D Poisson solver implemented with the discontinuous Galerkin (DG) method, developed and validated in our previous reports [36,37,38]. Based on the simulation results, we compared the potential and electric field distribution on different 2D concentric electrodes, where the distance between the inner electrode and outer ring varied between 50 μm, 200 μm, and 1000 μm. Further, we studied the potential and electric field distribution in the case of 3D electrodes and compared the results with the values obtained for 2D devices. The computation domain is a 2800 μm × 2800 μm × 750 μm box which is divided into two parts, where the upper part (500 μm) is filled with the solution (relative permittivity is 79) and the lower part (250 μm) is the silicon wafer substrate (relative permittivity is 11.68). The electrode is located on top of the substrate, but its thickness barely affects the overall field distribution and was neglected due to its extremely small value (200 nm) in comparison with the simulation domain size.

In the simulations, the inner electrode is given a fixed voltage of 4 V, and the voltage on the outer ring is set to 0 V by applying the Dirichlet boundary condition, which is the same voltage value as that used in the experiment. The Neumann boundary condition is applied on all the truncation boundaries to imitate the realistic potential variation on the boundaries. With electric field simulation results, the microfluidic motions produced by the electrodes due to the application of ac-field can be explained in a more straightforward and intuitive way.

### 2.4. Electrochemical Measurements

Cyclic voltammetry measurements were performed at different scan rates (10 mV/s to 100 mV/s) using inner 2D and 3D electrodes as working electrodes. A silver wire was used as a reference electrode. A gold wire was used as the counter electrode to complete the three-electrode system. The electrochemical measurements were performed using a solution containing 1 mM potassium hexacyanoferrate(II) trihydrate (Sigma-Aldrich, St. Louis, MI, USA) and 0.1 M KCl (Sigma-Aldrich) dissolved in ultrapure water (resistivity, 18.2 MΩ·cm). The electrochemical measurements were carried out using a USB potentiostat (Sensit Smart, PalmSens) operated by PSTrace5 software. The potential range used was −0.1 V to +0.5 V.

## 3. Results and Discussion

### 3.1. 2D Ac-EHD Devices

**Figure 3:**
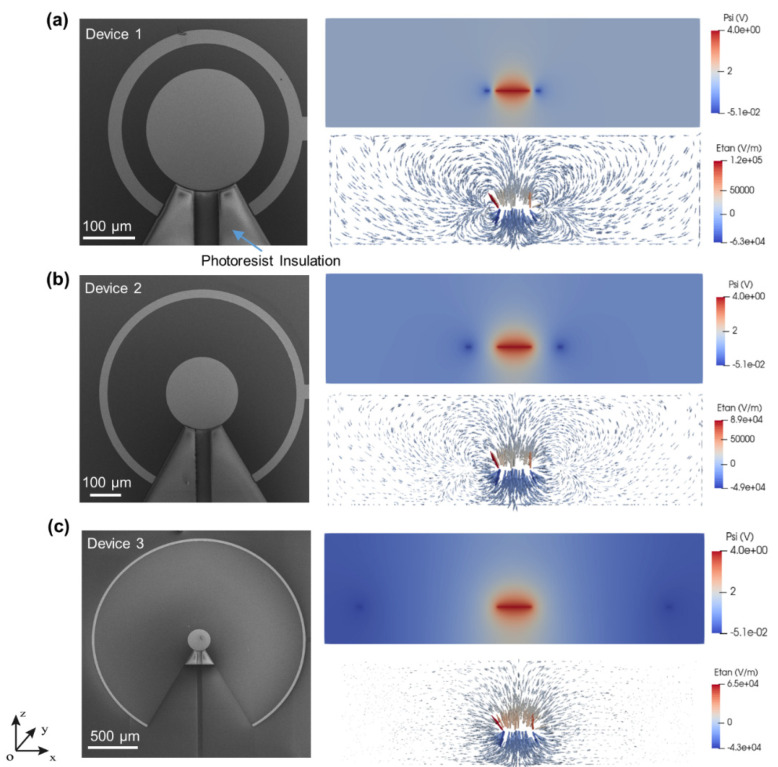
(**a**–**c**) (left column) shows the SEM images of 2D devices. The right column shows the electric potential and tangential electric field distribution of respective 2D devices on the cross-section of the -xoz plane.

Figure 3 shows the SEM images of the three different 2D ac-EHD devices along with the electric potential and tangential electric field distribution of 2D devices on a cross-section of -xoz plane. SEM images clearly show the fabrication of three different 2D devices. The inner electrode diameter (i.e., 250 µm) and outer electrode width (i.e., 30 µm) are the same in all devices. Device 1, device 2, and device 3 have spacings of 50 µm, 200 µm, and 1000 µm, respectively. The arrow in Figure 3a shows the photoresist insulation to define the gold electrode.

Previous studies using these ac-EHD devices focus mainly on the functionalization and application of the devices [16,18,39]. In the current study, detailed electric field simulations have been carried out to understand the effect of electrode spacing on the devices’ performance. From the simulation results (Figure 3), we can see that for all three devices, the tangential field reaches its maximum at the edge of the inner electrode, where the potential changes fastest, and drops rapidly as it gets away from the inner electrode edge towards the outer ring. Due to the smallest distance between the inner and outer electrodes, device 1 (Figure 3a) with spacing 50 µm has the largest tangential electric field peak value on the edge, while the lowest peak value among these three devices was found in device 3, with a spacing of 1000 µm.

**Figure 4:**
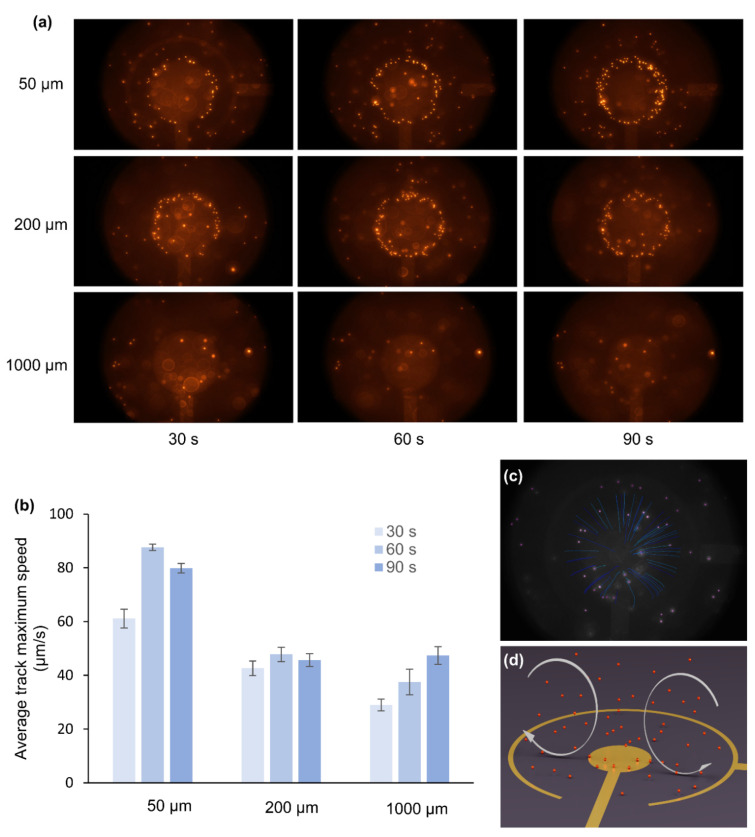
(**a**) Snapshots taken after 90 s of device operation in each case. (**b**) Average maximum speed in 2D devices calculated in intervals of 30, 60, and 90 s. The error bars show the calculation of the Average maximum speed from three ac-EHD devices in each case. (**c**) Snapshot from the particle tracking video obtained using NIH ImageJ software and Trackmate plugin (Video S4, Supplementary Materials). (**d**) Schematic of the ac-EHD driven fluid flow.

Upon applying an ac electric field (Figure 2), fluid motion was observed in all three different geometries (Figure 4 and Videos S1–S3, Supplementary Materials). The red fluorescent beads, dispersed in a buffer solution, are dragged by fluid viscosity and allow the fluid motion characterization on these devices using a fluorescence microscope. Figure 4a shows the snapshots taken from the videos of device 1, 2, and 3 after 90 s of application of ac-EHD signal (Videos S1–S3, Supplementary Materials). The ac-signal parameters used were V_pp_ = 4 V and *f* = 100 Hz, which previously showed the optimum performance for these devices [18].

It can be seen that after 90 s of operation, fluorescent beads accumulated at the edges of device 1 and device 2. However, this accumulation of beads can hardly be found in the case of device 3. The beads accumulate at the edges of inner electrodes in devices 1 and 2 to compensate for the large electric field (Figure 3a,b). However, compared to devices 1 and 2, the electric field varies smoothly in the entire region in device 3 (Figure 3c); hence, less or no attachment of beads at the edges of the inner electrode. The phenomenon observed in these experiments correlated with the previous reports [14,40] and indicates the dominance of the dielectrophoresis, which seems to be minimal for the device with the largest distance between electrodes. Taking advantage of this benefit, device 3 will be more suitable for many sensor applications [10]. Furthermore, Figure 4b shows that device 1 has the highest average track maximum speed, while device 3 has the lowest speed since the dielectrophoresis (DEP) force on a particle is proportional to $\left|\nabla E^{2}\right|$ [41]. Previously, the beads’ average speed over the total period of time was used to characterize the fluid motion. In this study we adopted a different approach; ac-EHD was performed for 90 s at 4 V and 100 Hz for each device, and their average maximum speed was analyzed on intervals of 30 s (Figure 4b). This analysis gave an entirely new perspective about the fluid motion in the ac-EHD devices. Figure 4b shows that in device 1 (spacing of 50 µm), the fluorescent beads’ speed corresponding to the fluid motion increased in the first 30 s and reached its maximum value at 60 s and then further decreased at 90 s. The decrease in the fluid flow may be due to the beads’ accumulation at the edges of the electrode, which partly cover the electrode surface, resulting in the slow fluid motion. In device 2 (spacing of 200 µm), the speed of the beads was almost half the maximum speed attained by device 1; however, the fluid motion remained almost steady except for a slight drop in the speed at 90 s interval. In device 3 (spacing of 1000 µm), the fluid velocity increased steadily throughout the 90-s video and attained half the speed compared to device 1. Figure 4c,d show the snapshot from the particle tracking video (Video S4, Supplementary Materials) and schematic illustration of the fluid movement due to ac-EHD, respectively. The tracking of the fluorescent beads gave information about the movement of the fluid, as illustrated in Figure 4d. When the ac-field is applied on the electrodes, the product between the induced charges in the electrical double layer and the electric field’s tangential component results in a force perpendicular to the electrode [18,42]. Due to the electrodes’ asymmetry, larger forces are generated in the outer ring, dictating the fluid motion from the inner to the outer electrode [18]. The ac-EHD fluid flow can also be tuned by changing the electric field strength by selecting different amplitude (V_pp_) and frequency (*f*) values [18]. In conclusion, device 1 gave the highest fluid mixing; however, the efficacy of fluid mixing decreased after 60 s. On the other hand, the fluid velocity increased steadily in case of device 3, indicating that the electrode spacing plays a crucial role both in the fluid mixing and emergence of the dielectrophoresis effect observed in device 1 and 2.

### 3.2. 3D Ac-EHD Device

**Figure 5:**
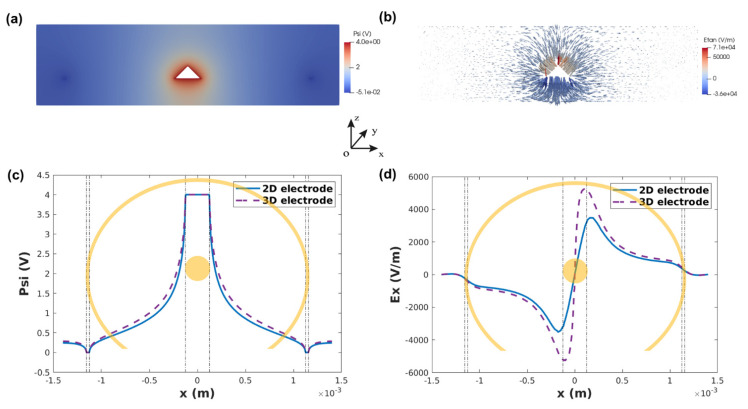
(**a**) Potential distribution and (**b**) tangential electric field distribution on the cross-section of -xoz plane. (**c**) 2D/3D potential comparison along *x* axis at z = 0 µm and (**d**) electric field comparison along *x* axis at z = 150 µm.

Device 3 showed a homogeneous distribution of the electric field and fluid motion compared to devices 1 and 2. Therefore, we chose this device design for the electric field simulation and fabrication of 3D devices. Figure 5a,b show the electric potential and tangential electric field distributions on the cross-section of the -xoz plane for a 3D electrode, where we can see the electric field peak on the edge of the inner electrode and a smooth transition towards the outer electrode, similarly to what was observed for 2D devices. To compare the performance of 2D and 3D electrodes, the potential distribution (at z = 0 µm) and electric field distribution (at z = 150 µm) along the *x*-axis are plotted in Figure 5c,d. It is clear that the 3D electrode has higher peak values of the electric field, and the electric field in the entire solution is higher than 2D electrodes. Therefore, 3D electrodes could provide better fluid micromixing leading to better sensing performance than 2D electrode with similar spacing (1000 µm), while maintaining minimum dielectro-phoresis effect.

**Figure 6:**
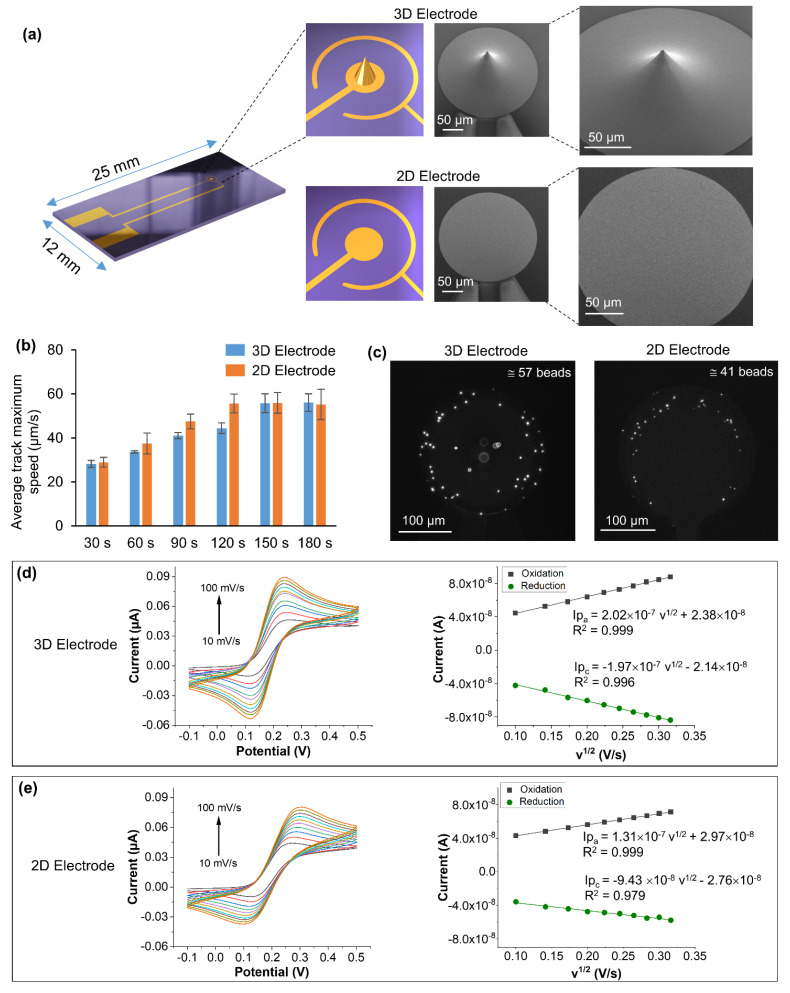
(**a**) Shows the schematic and SEM images of the 3D and 2D electrodes (inner electrodes). A single tip was formed in the middle of the inner electrode compared to the 2D electrode. (**b**) Comparison of the average maximum speed of 3D and 2D devices calculated in the intervals of 30 to 180 s. (**c**) Fluorescence images of 3D and 2D electrodes after the binding of streptavidin fluorescent beads onto the biotin-functionalized electrodes. The ac-EHD parameters used were 100 Hz, 4Vpp. (**d**,**e**, left) cyclic voltammograms were obtained using 0.1 M KCl containing 1 mM potassium ferrocyanide for 3D and 2D electrodes at different scan rates (10 mV/s to 100 mV/s). (**d**,**e**, right) present the plot current (I) vs. v^1/2^ for 3D and 2D electrodes, respectively.

Figure 6a shows SEM images of the 3D electrode fabricated in the case of device 3, along with a comparison with the 2D electrode. The illustrations and SEM images of 3D and 2D electrodes show a clear difference between the two electrodes confirming the fabrication of the 3D electrode. A single tip structure of ~110 µm height with a base of ~120 µm was fabricated at the center of the inner electrode (diameter 250 µm). Figure 6b shows the average maximum speed of the beads in the case of 3D electrode (Video S5, Supplementary Materials) compared to the 2D device (both have 1000 µm spacing between the inner and outer electrodes). It can be seen that the average maximum speed of the beads increased and becomes constant both in the case of 2D and 3D after 150 s.

Figure 6c shows the attachment of streptavidin fluorescent beads onto the biotin functionalized 2D and 3D devices. It is clear that after 180 s, more streptavidin beads were attached to the 3D electrodes compared to the 2D electrode under the same condition (100 Hz, 4V_pp_). In the case of the 3D electrode (Figure 6c), some of the beads appeared out of focus, especially the ones attached to the top of the tip, due to the limitation of the depth of focus in optical microscopy. Both the electrodes were functionalized with biotin under the same conditions; however, the 3D electrode showed a higher number of beads attached to the electrode surface than the 2D electrode (~40% higher efficiency of capturing specific beads). This can be explained by correlating the higher peak values of the electric field obtained in the 3D electrode (Figure 5c,d) due to the large surface area compared to the 2D electrode, resulting in a higher number of beads attached to the 3D electrode surface. Moreover, due to the large surface area, the fluid motion volume is more significant in the 3D electrode than the 2D electrode contributing to the attachment of more beads under the same conditions. Furthermore, to verify these results and to demonstrate the application potential of 3D electrodes for bioassays, electrochemically active surface area was calculated [43] both for 2D and 3D electrodes by performing cyclic voltammetry measurements (Figure 6d,e) and using the Randles-Sevick equation.

Ip_a_ = 2.69 × 10^5^ *n*^3/2^
*A D*^1/2^
*C* v^1/2^ (1)

where Ip_a_ is the anodic peak current (*A*), *n* is the number of electrons, *A* is the active surface area of the electrode (cm^2^), *D* is diffusion coefficient (*D* = 6.70 × 10^−6^ cm^2^·s^−1^), *C* is the concentration in mol·cm^−3^, and v is scan rate (V·s^−1^). The electrochemically active surface area of 3D and 2D electrodes was calculated from Equations (2) and (3) obtained from Figure 6d,e, and by using the Randles-Sevick equation.

Ip_a_ = 2.02 × 10^−7^ v^1/2^ + 2.38 × 10^−8^ (2)

Ip_a_ = 1.31 × 10^−7^ v^1/2^ + 2.97 × 10^−8^ (3)

The electrochemically active surface area of 3D and 2D electrodes was found to be 0.112 cm^2^ and 0.073 cm^2^, respectively. The higher value of the electrochemically active surface area of the 3D electrode compared to the 2D electrode correlates with the increase in the capture efficiency of the 3D electrode. These preliminary results indicate that 3D ac-EHD devices could help develop more efficient and sensitive biological assays using ac-EHD devices in the future.

## 4. Conclusions

We showed the first proof of concept of concentric 3D electrodes-based ac-EHD devices. To characterize the fluid motion due to 2D and 3D ac-EHD devices, we adopted a new approach and analyzed the average maximum speed of beads in intervals rather than the overall average maximum speed, which gave insightful information about the behavior of different 2D and 3D devices. Further-more, we show that the bead attachment at the edges of the inner electrode due to the dominant dielectrophoretic effect is strongly dependent on the distance between inner and outer electrodes in the case of ac-EHD devices. 3D ac-EHD devices provided a larger surface area, resulting in better fluid micromixing and attachment of large number of streptavidin beads at the electrode surface compared to 2D devices. These results showed the potential of 3D ac-EHD devices for application in biological assays in the future.

## Data Availability

Data is contained within the article or Supplementary Material.

## Supplementary Materials

The following supporting information can be downloaded at: https://www.mdpi.com/article/10.3390/bios12040215/s1, Video S1: Device 1; Video S2; Device 2; Video S3: Device 3; Video S4: Bead Tracking; Video S5: 3D electrode.
